# NIS2+™, an effective blood-based test for the diagnosis of at-risk nonalcoholic steatohepatitis in adults 65 years and older

**DOI:** 10.1097/HC9.0000000000000223

**Published:** 2023-08-09

**Authors:** Arun J. Sanyal, Jérémy Magnanensi, Zouher Majd, Christian Rosenquist, Delphis M. Vera, James P. Almas, Margery A. Connelly

**Affiliations:** 1 Stravitz-Sanyal Institute for Liver Disease and Metabolic Health, Virginia Commonwealth University School of Medicine, Richmond, Virginia, USA; 2 Biology Department Genfit SA, Loos, France; 3 Diagnostics Quality, Labcorp, Durham, North Carolina, USA; 4 Digital Innovation Group, Labcorp, Burlington, North Carolina, USA; 5 Diagnostics R&D, Labcorp, Morrisville, North Carolina, USA

## Abstract

Older patients are at increased risk for at-risk NASH, defined as NASH with NAFLD activity scores (NAS) ≥4 and significant fibrosis (F ≥ 2). The aim of this study was to compare the performance of 2 new blood tests, NIS4^®^ and NIS2+™, with FIB-4, NFS, ELF™, and alanine aminotransferase (ALT) for the diagnosis of at-risk NASH in a cohort of patients aged ≥65 years.

**Methods:**

The clinical performance of multiple blood-based tests was assessed for their ability to detect at-risk NASH using the RESOLVE-IT diag cohort, a large population of patients with metabolic risk who were screened for potential inclusion in the RESOLVE-IT phase 3 trial.

**Results:**

The study cohort (n = 2053) included patients with the full histological spectrum of NAFLD, with patients having liver fibrosis stages F0–4 and NAS scores 0–8. NIS4^®^ and NIS2+™ showed similar assay performance in patients who were <65 versus ≥65 years of age (AUROC = 0.80 vs. 0.78, *p* = 0.47; 0.81 vs. 0.83 *p* = 0.45, respectively) for the identification of at-risk NASH. In patients ≥65 (n = 410), NIS2+™ exhibited the highest AUROC compared to NIS4^®^, FIB-4, NFS, ELF™, and ALT (AUROC = 0.83 vs. 0.78, 0.68, 0.58, 0.69, 0.74, respectively; all *p* ≤ 0.0009). For NIS2+™, the sensitivity and NPV for ruling-out at-risk NASH at the 0.46 cutoff were 90.2% and 86.0%, and the specificity and PPV for ruling-in at-risk NASH at the 0.68 cutoff were81.1% and 76.3%, respectively.

**Conclusions:**

The clinical performance of NIS2+™ was superior for the diagnosis of at-risk NASH in patients ≥65 years of age. These data support the clinical value of this blood-based test for the diagnosis of at-risk NASH in older adults.

## INTRODUCTION

NAFLD is becoming the leading cause of chronic liver disease worldwide.^1–3^ In the United States alone, the prevalence of NAFLD and NASH has increased over the past decade, culminating in an estimated prevalence of 25%–30% and 2.5%–5%, respectively.^1,4–8^ The most common diseases associated with NAFLD and NASH are cardiometabolic diseases such as type 2 diabetes (T2D), obesity, hypertension, and dyslipidemia, which are also growing in prevalence in the United States and globally.^2,9^ It has been estimated that up to 20% of adults with NAFLD have NASH, and NASH has become the leading cause of chronic liver disease.^10,11^ At-risk NASH, which is defined as having NASH with NAFLD activity scores (NAS) ≥4 and significant liver fibrosis (F ≥ 2), based on histological scoring of liver biopsy, is associated with increased risk of disease progression as well as liver-related and all-cause mortality.^12–15^ Recently, the prevalence of at-risk NASH was shown to be about 4.4% in the general United States population and up to 18.3% in patients with T2D.^7^ Along with the increased prevalence in the United States, NASH has become the second leading cause of end-stage liver disease and the fastest-growing etiology of HCC and need for a liver transplant.^1,16–20^

Older adults, in particular those on Medicare, have an even higher risk for NAFLD-mediated and NASH-mediated progression to cirrhosis, acute on chronic liver failure, liver transplantation, and HCC.^21–24^ A recent article reported that NAFLD is the leading etiology of HCC among Medicare recipients (2011–2015), accounting for 35.6% of the HCC cases.^24^ In turn, among Medicare recipients with HCC, NAFLD was the most common cause of mortality.^25^ Compared to other etiologies, NAFLD was associated with lower surveillance for HCC, reduced early detection, and poorer overall survival.^21,24^ In addition, NASH exceeded all other causes of liver transplantation in Medicare patients in 2019 and, along with alcohol-associated hepatitis, is considered one of the most rapidly increasing etiologies of liver transplantation.^23,26^ The proportion of patients with NASH among elderly liver transplant candidates increased from 13% in 2002–2005 to 39% in 2018–2020.^23^ Moreover, a recent study in Medicare recipients (2007–2015) showed that older patients were likely to be diagnosed with cirrhosis before being diagnosed with NASH and that later-stage diagnosis of patients with NASH led to a substantial increase in health care resource utilization and costs.^27^ Therefore, it is critical to identify older adults with NASH in order to treat and reduce the progression to cirrhosis, HCC, and the need for a liver transplant.

NIS4^®^ is a blood test that combines the results of 4 biomarkers (miR-34a-5p, YKL-40, α2-macroglobulin, and glycated hemoglobin [HbA1c]) into a single score that aids in the diagnosis of at-risk NASH.^28^ In the noninvasive metabolic liver disease assessment study, which was conducted by the Foundation for the National Institutes of Health Biomarkers Consortium and the NASH Clinical Research Network, NIS4^®^ achieved significantly higher performance than alanine aminotransferase (ALT) and OWLiver for the diagnosis of patients with NASH.^29,30^ For the diagnosis of significant fibrosis (F≥2), NIS4^®^ outperformed Fibrosis-4 (FIB-4), Enhanced Liver Fibrosis test (ELF™), and Pro-Peptide of Type III Collagen (Pro-C3), albeit the latter tests were originally designed for the detection of advanced liver fibrosis (stage F ≥ 3).^30^ Importantly, NIS4^®^ was significantly better than ALT and FIB-4 for the identification of at-risk NASH.^29,30^ Recently, NIS2+™ was developed as an optimization of the NIS4^®^ technology by removing α2-macroglobulin and HbA1c from the equation and combining the results for miR-34a-5p and YKL-40 with a sex correction for miR-34a-5p, in order to robustly detect at-risk NASH, irrespective of patient characteristics such as age, sex, BMI, or T2D status.^31^ Previously published data showed that NIS2+™ had a high overall clinical performance for the detection of at-risk NASH, achieving an area under the receiver operating characteristic curve (AUROC) of 0.81 in a large study population.^31^

For the purposes of reimbursement, the Centers for Medicare & Medicaid Services in the United States requires peer-reviewed, published data showing that tests that will be used for patient management have good assay performance in patients who are ≥65 years of age. To date, the robustness of the NIS4^®^ and NIS2+™ tests has not been assessed specifically in patients who are ≥65 years of age. Therefore, the first aim of this study was to perform a retrospective, cross-sectional analysis assessing the performance of NIS4^®^ and NIS2+™ in patients <65 and ≥65 years of age in order to ensure the 2 tests detect at-risk NASH equivalently in both younger and older individuals. The second aim was to compare NIS4^®^, NIS2+™, FIB-4, NAFLD fibrosis score (NFS), ELF™, and ALT in a cohort of patients ≥65 years of age in order to determine which test had the highest overall performance for detecting at-risk NASH in elderly patients who would benefit from intensive lifestyle or therapeutic interventions.

## METHODS

### Study population

The RESOLVE-IT diag cohort consisted of patients screened for, and who may or may not have eventually been enrolled in, the RESOLVE-IT trial (NCT02704403), a multicenter, randomized, double-blind, placebo-controlled phase 3 trial designed to evaluate the efficacy and safety of elafibranor in patients with NASH and liver fibrosis (n > 5000 patients).^28^ Including patients who were screened for, but may not have been included in, the RESOLVE-IT trial enabled the current study to include subjects that were balanced across the histological endpoints of NASH and liver fibrosis. It also empowered the study to include a larger number of patients ≥65 years of age compared to the RESOLVE-IT trial alone. Patients with available biopsy results and with full data for NIS4^®^ (Genfit SA), NIS2+™ (Genfit SA), FIB-4, NFS, ALT, and ELF™ (Siemens Healthineers) at baseline were included in this analysis. To ensure the relevance of the biomarker concentrations versus histology, only patients with a gap of ≤90 days between biopsy and blood sample dates were retained. This process of selection led to a study cohort of n = 2053 patients, which was then divided into 2 groups: <65 (n = 1643) and ≥65 (n = 410) years of age. Patients in the study cohort were recruited at 249 global centers (including the United States and Europe) and screened between June 2016 and March 2020. The percentage of patients from the United States in the total study cohort was 46% (947/2053) and in those who were ≥65 years of age was 50% (205/410), the latter representing the Medicare population. The inclusion criteria for initial screening of the RESOLVE-IT diag cohort included patients 18–75 years of age with one or more metabolic disease risk factors (eg, T2D, obesity, dyslipidemia, and hypertension). Patients were excluded if daily alcohol consumption was greater than 2 drink units per day (20 g) in women and 3 drink units per day (30 g) in men, hepatosteatosis was due to secondary causes, or they had other chronic liver diseases. During the screening stage, clinical data, blood samples, and liver biopsy results were collected for all patients included in this study. Liver biopsies were scored based on the NASH Clinical Research Network classification system. Histological scoring was performed by a single trained pathologist at Hôpital Beaujon (Paris, France), who was blinded to the clinical and biochemistry data. All research for this study was conducted in accordance with both the Declarations of Helsinki and Istanbul. The clinical study protocol was approved in all countries by national authorities and institutional ethics review committees, and all patients provided written informed consent for participation in the trial and collection of blood samples for biomarker research. The names of the IRB committees and the approval numbers are available upon request.

### Clinical characteristics and laboratory tests

Demographic, medical history, and anthropometric characteristics, including body weight, height, age, sex, and BMI, were collected at clinical sites from each patients’ medical record. Blood samples were collected after fasting for at least 12 hour. Biochemical analyses were performed using blood samples collected at a median time and mean time of 25 and 16 days, respectively, after biopsy. Hematology and biochemical analyses, including α2-macroglobulin and HbA1c, were conducted by BARC-Europe (Ghent, Belgium). All biochemical analyses were performed using commercially available kits or reagents. YKL-40 (DC3L10 kit; Bio-Techne, Minneapolis, MN) and miR-34a-5p assays were performed in the laboratory at Genfit SA (Loos, France). Details of the miR-34a-5p assay and the development of the NIS4^®^ and NIS2+™ algorithms, including the determinations of the cutoffs, were reported.^28,31^ Neither vibration-controlled transient elastography (VCTE) FibroScan nor magnetic resonance elastography results were available for many of the participants; therefore, VCTE results were not used for comparison purposes. Patients with at-risk NASH were defined as those having NASH with NAS ≥ 4 and liver fibrosis stage F≥2.

### Test equations

The following previously published equations were used in this study^28,31–34^:

NIS4^®^ score = e^*y*^ / (1 + e^*y*^), where *y* = β_0_ + β_1_ * log_10_[miR-34a-5p (Fold)] + β_2_ * [A2M (g/L)] + β_3_ * log_10_[YKL-40 (ng/mL)] + β_4_ * [HbA1c (%)]

NIS2+™ score = e^*y*^ / (1 + e^*y*^), where *y* = β_0_ + β_1_ * log_10_[miR-34a-5p (Fold)] + β_2_ * log_10_[YKL-40 (ng/mL)] + β_3_ * sex + β_4_ * log_10_[miR-34a-5p (Fold)] * sex (sex coded 0 if female, 1 if male)

FIB-4 score = [Age (y) × AST (U/L)] / [PLT (10^9^/L) × ALT (U/L)^1/2^]

NFS score = −1.675 + 0.037 × age (y) + 0.094 × BMI (kg/m^2^) + 1.13 × IFG/diabetes (yes = 1, no = 0) + 0.99 × AST/ALT ratio − 0.013 × platelet count (×10^9^/L) − 0.66 × albumin (g/dL)

ELF™ score = 2.494 + 0.846 In (C_HA_) + 0.735 In (C_PIIINP_) + 0.391 In (C_TIMP-1_)

### Statistical analysis

Descriptive statistics were generated for baseline characteristics of the full cohort, and patients aged <65 and ≥65 years of age were compared using appropriate 2-sample tests (Student *t* test for comparison of means, Wilcoxon test for comparison of medians, and χ^2^ test for comparison of percentages). Some biomarkers were associated with heavy-tailed distributions, so that median (IQR) values were reported instead of mean ± SD. The overall diagnostic performance of different blood tests for the detection of at-risk NASH were assessed using area AUROC values and associated 95% CIs obtained by using 1000 bootstrap samples. Differences between AUROC values for NIS2+™ versus NIS4^®^ and other noninvasive tests were assessed using paired Delong tests (pROC R package, version 1.18.0; the R Foundation, Vienna, Austria). Published low and high cutoffs for each noninvasive test were used to derive clinical performance parameters such as sensitivity, specificity, negative predictive value (NPV), and positive predictive value (PPV) for ruling-in and ruling-out at-risk NASH.^28,31–33,35^ Upper limit normal values for ALT of 33 IU/L for women and 41 IU/L for men have been used as single cutoff values for ruling-in and ruling-out at-risk NASH.

The Youden Index statistic, another measure of a test’s effectiveness, was defined as the sum of specificity and sensitivity minus 1, which could be used to derive an optimal cutoff for a biomarker, also called Youden cutoff, in that it would maximize the Youden Index statistic. The Youden Index cutoffs were determined using the full study cohort (n = 2053) for the detection of at-risk NASH in order to provide a more reliable comparison of sensitivity, specificity, NPV, and PPV values among the tests for the same endpoint instead of those published for the detection of advanced fibrosis regarding FIB-4, NFS, and ELF™.

Sensitivity and specificity were compared using McNemar tests, and PPV and NPV were compared using Generalized Score tests (DTComPair R package, version 1.0.3; the R Foundation, Vienna, Austria). All statistics were derived with 95% confidence limits calculated using the Wilson score interval with correction for continuity (DescTools R package, version 0.99.44; the R Foundation, Vienna, Austria). A *p*-value < 0.05 was regarded as statistically significant for the analysis of the results. The intermediate (moderate risk) zone was defined as the range of scores between the low and high cutoffs. The proportion of patients within the intermediate (moderate risk) zone was compared using McNemar tests for equality of proportions with continuity correction.

## RESULTS

### Clinical characteristics of the study population

A comparison of the subpopulation of study participants who were ≥65 versus those who were <65 years of age based on baseline demographics, clinical characteristics, and laboratory results is provided in Table 1. Patients who were ≥65 were more likely to have T2D, dyslipidemia, arterial hypertension, lower BMI, and obesity compared to those who were < 65 years. They were also more likely to have a higher aspartate aminotransferase (AST)/ALT ratio, fasting plasma glucose (FPG), HbA1c, and lower ALT, total cholesterol, and platelet count. While the median model for end-stage liver disease (MELD) score was similar, more patients who were ≥65 were in the higher MELD score classes. While the percentages of adults with NAFLD, NASH, and at-risk NASH were similar between adults who were <65 versus ≥65 years, patients who were ≥65 years had a higher mean liver fibrosis stage compared to those who were <65 years (2.16 vs. 1.79, *p*<0.0001) and had a lower mean NAS (3.84 vs. 4.17, *p* = 0026) based on liver biopsy results (Table 1).

**TABLE 1:** Baseline demographics and clinical characteristics for the RESOLVE-IT diag study population

	n	All data	n	<65 y	n	≥65 y	*p*
% male	2053	1275 (62)	1643	1020 (62)	410	255 (1020)	1
Age (y)	2053	54 ± 12	1643	50 ± 10	410	69 ± 3	<0.01
Ethnicity	2031	—	1626	—	405	—	<0.0001
White	—	58	—	56	—	63	—
Hispanic	—	33	—	34	—	29	—
Asian	—	3	—	3	—	3	—
Black	—	3	—	3	—	4	—
Other	—	3	—	4	—	1	—
Type 2 diabetes	2053	857 (42)	1643	649 (40)	410	208 (51)	<0.0001
Dyslipidemia	2053	981 (48)	1643	737 (45)	410	244 (60)	<0.0001
Arterial hypertension	2053	1150 (56)	1643	852 (52)	410	298 (73)	<0.0001
BMI (kg/m^2^)	2053	33.5 ± 6.1	1643	33.9 ± 6.2	410	31.8 ± 5.1	<0.0001
Obese^a^, n (%)	2053	1439 (70)	1643	1190 (72)	410	249 (61)	<0.0001
Laboratory measures							
AST (IU/L)	2053	32 (23–48)	1643	33 (23–49)	410	31 (23–46)	0.19
ALT (IU/L)	2053	44 (29–69)	1643	46 (30–72)	410	38 (25–58)	<0.0001
AST/ALT ratio	2053	0.75 (0.61–0.95)	1643	0.72 (0.59–0.91)	410	0.84 (0.72–1.06)	<0.0001
GGT (IU/L)	2053	44 (29–77)	1643	44 (29–76)	410	45.5 (26–83)	0.98
ALP (IU/L)	2052	79 (64–99)	1642	79 (64–99)	410	79 (63–99)	0.97
FPG (mmol/L)	2050	5.44 (4.88–6.57)	1640	5.38 (4.83–6.49)	410	5.8 (5.11–6.88)	<0.0001
HbA1c (%)	2053	6.18 ± 1.01	1643	6.14 ± 1.02	410	6.32 ± 0.97	0.0015
Triglycerides (mmol/L)	2053	1.67 (1.24–2.30)	1643	1.68 (1.24–2.34)	410	1.63 (1.25–2.15)	0.10
Total cholesterol (mmol/L)	2051	4.74 ± 1.16	1642	4.79 ± 1.16	409	4.51 ± 1.12	<0.0001
HDL cholesterol (mmol/L)	955	1.14 (0.96–1.35)	774	1.11 (0.93–1.32)	181	1.22 (1.01–1.45)	0.0001
LDL cholesterol (mmol/L)	912	2.69 ± 0.98	734	2.73 ± 0.97	178	2.5 ± 0.97	0.0039
Platelet (10^9^/L)	2053	238 ± 68	1643	243 ± 67	410	218 ± 68	<0.0001
Scores							
MELD	2040	8 (7–9)	1634	8 (7–9)	406	8 (7–9)	0.0013
MELD score by class							
6–7	2040	40	1634	41	406	37	<0.0001
8–10	—	54	—	54	—	55	—
11–15	—	5.4	—	4.8	—	8.1	—
16–20	—	0.2	—	0.2	—	0.2	—
NIS2+™	2053	0.57 (0.31–0.79)	1643	0.55 (0.31–0.79)	410	0.61 (0.37–0.79)	0.026
NIS4^®^	2053	0.51 (0.28–0.75)	1643	0.47 (0.26–0.71)	410	0.64 (0.40–0.82)	<0.0001
FIB-4	2053	1.17 (0.80–1.71)	1643	1.04 (0.74–1.53)	410	1.71 (1.31–2.37)	<0.0001
NFS	2053	−1.3 ± 1.54	1643	−1.53 ± 1.53	410	−0.37 ± 1.2	<0.0001
ELF™	2053	9.45 ± 1.01	1643	9.31 ± 0.97	410	10.01 ± 0.96	<0.0001
Histology							
NAFLD	2053	1881 (92)	1643	1510 (92)	410	371 (90)	0.41
NASH	2053	1360 (66)	1643	1103 (67)	410	257 (63)	0.10
Patients with at-risk NASH	2053	945 (46)	1643	752 (46)	410	193 (47)	0.68
Liver fibrosis	2053	1.87 ± 1.17	1643	1.79 ± 1.17	410	2.16 ± 1.16	<0.0001
Liver fibrosis stage	2053	—	1643	—	410	—	<0.0001
0	—	15	—	16	—	11	—
1	—	26	—	27	—	19	—
2	—	24	—	25	—	21	—
3	—	29	—	26	—	41	—
4	—	6	—	6	—	8	—
NAS	2053	4.10 ± 2.00	1643	4.17 ± 2.00	410	3.84 ± 1.96	0.0026
NAS category	2053	—	1643	—	410	—	<0.0001
0–1	—	13	—	13	—	15	—
2–3	—	22	—	21	—	27	—
4–5	—	37	—	37	—	36	—
6–8	—	28	—	29	—	22	—

*Note:* Data are n (%) or mean ± SD or median (IQR).

Variables that were non-normally distributed were log-transformed and are reported as median (IQR). At-risk NASH was defined as NAS ≥ 4 and liver fibrosis stage ≥2.

a BMI≥30 kg/m^2^. *p*-values were computed using either χ^2^ tests for proportions comparisons, Student *t* tests for means comparisons, and Wilcoxon tests for median comparisons and represent the comparison between patients <65 vs. ≥65 years of age.

Abbreviations: ALP, alkaline phosphatase; ALT, alanine aminotransferase; AST, aspartate aminotransferase; ELF™, Enhanced Liver Fibrosis test; FPG, fasting plasma glucose; GGT, γ-glutamyl transferase; HBA1c, glycated hemoglobin; NAS, NAFLD activity score; NFS, NAFLD fibrosis score.

Within the ≥ 65 age subpopulation (n = 410), the prevalence of at-risk NASH was 47% (193/410), with a lower percentage of males in this group compared to those without at-risk NASH (50% vs. 73%, *p* < 0.0001) (Table 2). A higher percentage of the at-risk NASH group were white (67% vs. 59%, *p* = < 0.0001) and had T2D (59% vs. 44%, *p* = 0.0039) as well as higher measures of AST, ALT, γ-glutamyl transferase (GGT), FPG, HbA1c, triglycerides, and total cholesterol compared to the non-at-risk NASH population. There was no statistically significant difference in the median MELD score between older patients with at-risk NASH and those with NASH, although those with at-risk NASH tended to be in a higher MELD score class. In addition, among older at-risk NASH patients, 58% had liver fibrosis stage F3 and 11% were cirrhotic (F4) (Table 2).

**TABLE 2:** Baseline demographics and clinical characteristics for patients aged ≥65 years with or without at-risk NASH

	n	All data	n	Non-at-risk NASH	n	At-risk NASH	*p*
% male	410	255 (62)	217	159 (73)	193	96 (50)	<0.0001
Age (y)	410	69 ± 3	217	69 ± 3	193	69 ± 3	0.40
Ethnicity	405	—	216	—	189	—	<0.0001
White	—	63	—	59	—	67	—
Hispanic	—	29	—	32	—	25	—
Asian	—	3	—	2	—	4	—
Black	—	4	—	5	—	2	—
Other	—	1	—	1	—	2	—
Type 2 diabetes	410	208 (51)	217	95 (44)	193	113 (59)	0.0039
Dyslipidemia	410	244 (60)	217	123 (57)	193	121 (63)	0.26
Arterial hypertension	410	298 (73)	217	153 (71)	193	145 (75)	0.35
BMI (kg/m^2^)	410	31.8 ± 5.1	217	31.6 ± 5.0	193	32.1 ± 5.3	0.32
Obese^a^	410	249 (61)	217	126 (58)	193	123 (64)	0.28
Laboratory measures							
AST (IU/L)	410	31 (23–46)	217	26 (19–34)	193	40 (30–59)	<0.0001
ALT (IU/L)	410	38 (25–58)	217	30 (21–43)	193	50 (35–68)	<0.0001
AST/ALT ratio	410	0.84 (0.72–1.06)	217	0.85 (0.72–1.05)	193	0.84 (0.72–1.06)	0.86
GGT (IU/L)	410	45.5 (26–83)	217	32 (23–55)	193	63 (41–118)	<0.0001
ALP (IU/L)	410	79 (63–99)	217	76 (63–96)	193	83 (63–103)	0.06
FPG (mmol/L)	410	5.8 (5.11–6.88)	217	5.55 (4.94–6.57)	193	6.05 (4.27–7.37)	0.0001
HbA1c (%)	410	6.32 ± 0.97	217	6.19 ± 0.93	193	6.45 ± 1.00	0.0071
Triglycerides (mmol/L)	410	1.63 (1.25–2.15)	217	1.57 (1.23–2.00)	193	1.73 (1.30–2.31)	0.0173
Total cholesterol (mmol/L)	409	4.51 ± 1.12	216	4.35 ± 1.03	193	4.68 ± 1.20	0.0032
HDL cholesterol (mmol/L)	181	1.22 (1.01–1.45)	9	1.35 (1.11–1.41)	172	1.22 (0.99–1.45)	0.54
LDL cholesterol (mmol/L)	178	2.50 ± 0.97	9	2.66 ± 0.82	169	2.49 ± 0.98	0.56
Platelet (10^9^/L)	410	218 ± 68	217	223 ± 72	193	213 ± 62	0.12
Scores							
MELD	406	8 (7–9)	216	8 (7–9)	190	8 (7–9)	0.11
MELD score by class							
6–7	406	37	216	39	190	34	NA
8–10	—	55	—	54	—	56	—
11–15	—	8.1	—	6.9	—	9.5	—
16–20	—	0.2	—	0	—	0.5	—
NIS2+™	410	0.61 (0.37–0.79)	217	0.42 (0.23–0.61)	193	0.77 (0.62–0.87)	<0.0001
NIS4^®^	410	0.64 (0.40–0.82)	217	0.48 (0.27–1.70)	193	0.77 (0.62–0.87)	<0.0001
FIB-4	410	1.71 (1.31–2.37)	217	1.52 (1.21–1.98)	193	1.96 (1.53–2.80)	<0.0001
NFS	410	−0.37 ± 1.2	217	−0.53 ± 1.24	193	−0.19 ± 1.14	0.0042
ELF™	410	10.01 ± 0 .96	217	9.72 ± 0.83	193	10.35 ± 0.99	<0.0001
Histology							
NAFLD	410	371 (90)	217	178 (82)	193	193 (100)	<0.0001
NASH	410	257 (63)	217	64 (29)	193	193 (100)	<0.0001
Fibrosis	410	2.16 ± 1.16	217	1.59 ± 1.23	193	2.79 ± 0.62	<0.0001
Fibrosis by stage	410	—	217	—	193	—	NA
0	—	11	—	21	—	0	—
1	—	19	—	36	—	0	—
2	—	21	—	12	—	32	—
3	—	41	—	26	—	58	—
4	—	8	—	6	—	11	—
NAS	410	3.84 ± 1.96	217	2.41 ± 1.39	193	5.46 ± 1.05	< 0.0001
NAS by category	410	—	217	—	193	—	NA
0–1	—	15	—	28	—	0	—
2–3	—	27	—	51	—	0	—
4–5	—	36	—	21	—	53	—
6–8	—	22	—	1	—	47	—

*Note:* Data are n (%) or mean ± SD or median (IQR).

Variables that were non-normally distributed were log-transformed and are reported as median (IQR). At-risk NASH was defined as NAS ≥ 4 and liver fibrosis stage ≥2. Of note, 95% (of subjects defined as having at-risk NASH in the RESOLVE-IT diag cohort (all ages) had AST above 20 IU/L.

a BMI ≥30 kg/m^2^. *p*-values represent the comparison between those with and without at-risk NASH. *p*-values were computed using either χ^2^ tests for proportions comparisons, Student *t* tests for means comparisons or Wilcoxon tests for median comparisons.

Abbreviations: ALP, alkaline phosphatase; ALT, alanine aminotransferase; AST, aspartate aminotransferase; ELF™, Enhanced Liver Fibrosis test; FIB-4, fibrosis-4; FPG, fasting plasma glucose; GGT, γ-glutamyl transferase; HBA1c, glycated hemoglobin; MELD, model for end-stage liver disease; NAS, NAFLD activity score; NFS, NAFLD fibrosis score.

### Performance metrics for NIS4^®^ and NIS2+™ in patients <65 versus ≥65 years of age

For the detection of at-risk NASH, NIS4^®^ and NIS2+™ achieved similar AUROC values in those who were <65 versus ≥65 years (0.80 vs. 0.78, *p* = 0.47; 0.81 vs. 0.83, *p* = 0.45, respectively) (Table 3). For ruling-out at-risk NASH, the sensitivity and NPV for NIS4^®^ at the low cutoff point of 0.36 was higher in patients aged ≥65 compared to those in patients < 65 years old (95.3% and 89.8% vs. 84.0% and 80.9%, respectively). While comparable results on sensitivity and NPV were obtained for NIS2+™ at the low cutoff point of 0.46 in between these 2 age groups, it was noticed that the difference in performance between the subpopulations was reduced compared to those associated with NIS4^®^. While both NIS4^®^ and NIS2+™ exhibited a high specificity at the cutoff point for ruling-out at-risk NASH in patients <65 years of age (57.0% vs. 62.9%, respectively), NIS2+™ retained high specificity in the older age group while NIS4^®^ was associated with a decreased specificity (53.9% vs. 36.4%, respectively) (Table 3). For ruling-in at-risk NASH, the specificity and PPV for NIS4^®^ at the high cutoff point of 0.63 were lower in patients aged ≥ 65 compared with those in patients < 65 years old, while NIS2+™ achieved similar performance among both subgroups at the high cutoff point of 0.68. Both NIS4^®^ and NIS2+™ achieved good sensitivity for ruling-in at-risk NASH, in both age groups, with the performance ranging between 55.9% and 73.6%. However, while NIS2+™ exhibited similar sensitivities in both age groups (61.2% vs. 68.4%), NIS4^®^ exhibited a notable difference across the 2 subgroups (55.9% vs. 73.6%) (Table 3). Overall, while both tests achieved high performance in both age groups, NIS2+™ returned more robust/stable performance across age groups compared with NIS4^®^.

**TABLE 3:** Clinical performance of NIS4^®^ and NIS2+™ for the detection of at-risk NASH, by age group

	At-risk NASH endpoint			
	NIS4^®^		NIS2+™	
	<65 y	≥65 y	<65 y	≥65 y
AUROC	0.80 (0.78, 0.82)	0.78 (0.74, 0.82)	0.81 (0.79, 0.83)	0.83 (0.79, 0.87)
Rule-out (low risk)				
Low cutoff	<0.36	<0.36	<0.46	<0.46
Sensitivity	84.0 (81.2, 86.5)	95.3 (91.1, 97.7)	84.0 (81.2, 86.5)	90.2 (84.8, 93.8)
Specificity	57.0 (53.7, 60.3)	36.4 (30.1, 43.2)	62.9 (59.6, 66.0)	53.9 (47.0, 60.6)
NPV	80.9 (77.5, 83.8)	89.8 (81.0, 94.9)	82.4 (79.2, 85.1)	86.0 (78.8, 91.2)
True negative (TN)	508 (81)	79 (90)	560 (82)	117 (86)
False negative (FN)	120 (19)	9 (10)	120 (18)	19 (14)
Intermediate zone (moderate risk)	456 (28)	110 (27)	374 (23)	101 (25)
Rule-in (high risk)				
High cutoff	≥0.63	≥0.63	≥0.68	≥0.68
Sensitivity	55.9 (52.2, 59.4)	73.6 (66.7, 79.5)	61.2 (57.6, 64.7)	68.4 (61.3, 74.8)
Specificity	84.4 (81.8, 86.7)	67.7 (61.0, 73.8)	85.5 (83.0, 87.7)	81.1 (75.1, 86.0)
PPV	75.1 (71.3, 78.6)	67.0 (60.2, 73.2)	78.1 (74.5, 81.3)	76.3 (69.1, 82.3)
True positive (TP)	420 (75)	142 (67)	460 (78)	132 (76)
False positive (FP)	139 (25)	70 (33)	129 (22)	41 (24)

Data are either percentage (95% confidence limits) or n (%).

Abbreviations: AUROC, area under the receiver operating characteristics curve; NPV, negative predictive value; PPV, positive predictive value.

Similar data were computed for the identification of at-risk NASH with F ≥ 3 and F4 using NIS2+™ (Supplemental Table S1, http://links.lww.com/HC9/A427). Overall, NIS2+™ kept stable clinical performance across age groups when identifying these endpoints, and was notably associated with an AUROC of 0.79 for the identification of at-risk NASH F ≥ 3 in patients aged ≥65 years of age. The low prevalence of at-risk NASH patients with cirrhosis in both age groups (4.4% and 5.1%) induced the low PPV and high NPV achieved by NIS2+™, and limited the interpretation of clinical performance due to the low number of cases in each age group (n = 72 and 21). However, it was interesting to note that no more than 4% to 5% of these at-risk NASH patients with cirrhosis were wrongly ruled out by NIS2+™ while more than 76% were accurately ruled in for both age groups.

### Comparison of performance characteristics across noninvasive tests

Clinical performance was assessed for NIS4^®^, NIS2+™, FIB-4, NFS, ELF™, and ALT for identification of patients with at-risk NASH in patients who were ≥65 years of age. In this older adult population, the clinical performance based on the AUROC was significantly higher for NIS2+™ than for all other tests (Table 4). At the low cutoff points, the specificity and NPV values were significantly better for NIS2+™ than for all other tests, except for the NPV for NIS4^®^ and ELF™, and the specificity for ALT, which was higher than that for NIS2+™ (66.8% vs. 53.9%, *p* = 0.0003). The sensitivity for NIS2+™ at the low cutoff point was lower than that for NIS4^®^ and ELF™ and similar for FIB-4 and NFS, but was higher than that for ALT. Notably, while ELF™ exhibited a sensitivity of 100%, the specificity was only 0.5% as this test was developed for ruling out advanced fibrosis and not for ruling out at-risk NASH. At the high cut points, the PPV values were significantly better for NIS2+™ than for all other tests except for FIB-4. The specificity for NIS2+™ was better than that for NIS4^®^, ELF™, and ALT, similar to that for NFS, and lower than that for FIB-4 (81.1% vs. 88.5%, *p* = 0.021). The sensitivity for NIS2+™ was higher than that for FIB-4 and NFS and similar to all other tests. NIS2+™ exhibited the smallest intermediate or moderate risk zone compared to all other tests except for NIS4^®^ (25% vs. 27%, *p* = 0.46) (Table 4). There was no intermediate risk zone calculated for ALT as upper limit normal was used for the analysis as a single cutoff.

**TABLE 4:** Clinical performance of NIS2+™ versus other blood tests for the identification of at-risk NASH in patients aged ≥65 years

	NIS2+™	NIS4^®^	*p*	FIB-4	*p*	NFS	*p*	ELF™	*p*	ALT	*p*
AUROC	0.83 (0.79, 0.87)	0.78 (0.74, 0.82)	0.0009	0.68 (0.63, 0.73)	<0.0001	0.58 (0.52, 0.63)	<0.0001	0.69 (0.64, 0.74)	<0.0001	0.74 (0.69, 0.79)	<0.0001
Rule-out (low risk)											
Low cutoff	<0.46	<0.36	—	<1.30	—	<−1.46	—	<7.70	—	<ULN	—
n (%)	136 (33)	88 (21)	—	102 (25)	—	68 (17)	—	1 (0.2)	—	195 (48)	—
Sensitivity	90.2 (84.8, 93.8)	95.3 (91.1, 97.7)	0.0039	86.0 (80.1, 90.4)	0.17	87.0 (81.3, 91.3)	0.30	100.0 (97.6, 100.0)	< 0.0001	74.1 (67.2, 80.0)	< 0.0001
Specificity	53.9 (47.0, 60.6)	36.4 (30.1, 43.2)	< 0.0001	34.6 (28.3, 41.3)	< 0.0001	19.8 (14.9, 25.9)	< 0.0001	0.5 (0.0, 2.9)	< 0.0001	66.8 (60.1, 73.0)	0.0003
NPV	86.0 (78.8, 91.2)	89.8 (81.0, 94.9)	0.16	73.5 (63.7, 81.5)	0.0056	63.2 (50.6, 74.4)	0.0002	100.0 (5.5 , 100.0)	0.32	74.4 (67.5, 80.2)	0.0001
Intermediate zone (moderate risk)	101 (25)	110 (27)	0.46	226 (55)	<0.0001	270 (66)	<0.0001	182 (44)	<0.0001	0	—
Rule-in (high risk)											
High cutoff	≥0.68	≥0.63	—	≥2.67	—	≥0.68	—	≥9.80	—	≥ULN	—
n (%)	173 (42)	212 (52)	—	82 (20)	—	72 (18)	—	227 (55)	—	215 (52)	—
Sensitivity	68.4 (61.3, 74.8)	73.6 (66.7, 79.5)	0.08	29.5 (23.3, 36.6)	<0.0001	19.7 (14.5, 26.1)	<0.0001	68.4 (61.3, 74.8)	1	74.1 (67.2, 80.0)	0.13
Specificity	81.1 (75.1, 86.0)	67.7 (61.0, 73.8)	<0.0001	88.5 (83.3, 92.3)	0.021	84.3 (78.6, 88.8)	0.38	56.2 (49.3, 62.9)	<0.0001	66.8 (60.1, 73.0)	<0.0001
PPV	76.3 (69.1, 82.3)	67.0 (60.2, 73.2)	0.0001	69.5 (58.2, 78.9)	0.17	52.8 (40.7, 64.5)	0.0002	58.1 (51.4, 64.6)	<0.0001	66.5 (59.7, 72.7)	0.0006
Youden											
Cutoff	<0.60	<0.52	—	<1.28	—	<−1.48	—	<9.57	—	<42.5	—
n (%)	199 (48.5)	147 (35.9)	—	95 (23.2)	—	65 (15.9)	—	139 (33.9)	—	231 (56.3)	—
Sensitivity	79.8 (73.3, 85.1)	87.0 (81.3, 91.3)	0.0043	87.0 (81.3, 91.3)	0.027	88.6 (83.0, 92.6)	0.017	79.3 (72.7, 84.6)	0.89	64.2 (57.0, 70.9)	<0.0001
Specificity	73.7 (67.3, 79.3)	56.2 (49.3, 62.9)	<0.0001	32.3 (26.2, 39.0)	<0.0001	19.8 (14.9, 25.9)	<0.0001	45.6 (38.9, 52.5)	<0.0001	74.7 (68.2, 80.2)	0.77
NPV	80.4 (74.1, 85.5)	83.0 (75.7, 88.5)	0.28	73.7 (63.5, 81.9)	0.11	66.2 (53.3, 77.1)	0.019	71.2 (62.8, 78.4)	0.013	70.1 (63.7, 75.9)	<0.0001
PPV	73.0 (66.4, 78.7)	63.9 (57.7, 69.6)	<0.0001	53.3 (47.7, 58.9)	<0.0001	49.6 (44.2, 55.0)	<0.0001	56.5 (50.3, 62.4)	<0.0001	69.3 (61.9, 75.8)	0.17

*Note:* Data are n (%) or percentage (95% confidence limits).

ULN values for ALT are 33 IU/L for women, 41 IU/L for men.

Abbreviations: ALT, alanine aminotransferase; AUROC, area under the receiver operating characteristics curve; ELF™, Enhanced Liver Fibrosis test; FIB-4, fibrosis-4; NFS, NAFLD fibrosis score; NPV, negative predictive value; PPV, positive predictive value; ULN, upper limit normal.

**FIGURE 1:**
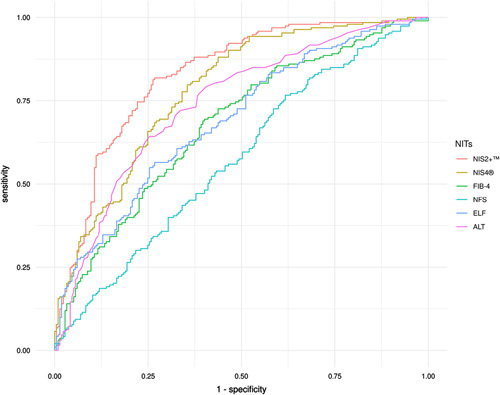
Comparison of AUROC values for NIS2+™, NIS4^®^, FIB-4, NFS, ELF™, and ALT scores in the RESOLVE-IT diag cohort. Abbreviations: ALT, alanine aminotransferase; AUROC, area under the receiver operating characteristic curve; ELF™, Enhanced Liver Fibrosis test; FIB-4, fibrosis-4; NFS, NAFLD fibrosis score.

Considering the Youden cutoff, NIS2+™ exhibited significantly higher PPV compared to all other tests except for ALT (73.0% vs. 69.3%, *p* = 0.17) and also exhibited a significantly higher NPV compared to all tests except for NIS4^®^ and FIB-4. Moreover, NIS2+™ had higher specificity to all tests except for ALT (73.7% vs. 74.7%, *p* = 0.77). The sensitivity for NIS2+™ was similar to or somewhat lower than that for all tests, except for ALT, where it was higher (79.8% vs. 64.2%, *p* < 0.0001) (Table 4). Notably, the Youden cutoff point for NIS2+™ was very close to the high cutoff point (0.60 vs. 0.68). Overall, NIS2+™ exhibited greater clinical performance when compared to NIS4^®^, FIB-4, NFS, ELF™, and ALT (Figure 1).

### Ability of NIS4^®^ and NIS2+™ to differentiate between NAS scores and liver fibrosis stages

**FIGURE 2:**
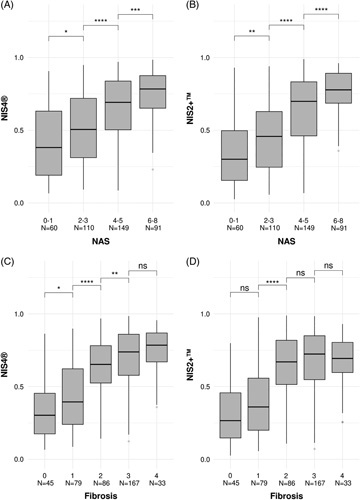
Box plots of mean ± SD for NIS4^®^ and NIS2+™ scores by subgroup of NAS (0–1, 2–3, 4–5, 6–8) and liver fibrosis stage (F0, F1, F2, F3, F3) based on histological analysis of liver biopsy results. (A) NIS4^®^ score by NAS, (B) NIS2+™ by NAS, (C) NIS4^®^ by liver fibrosis stage, (D) NIS2+™ by liver fibrosis stage. * = < 0.05, ** = <0.01, *** = <0.001, **** = <0.0001. Abbreviations: NAS, NAFLD activity score; ns, not significant.

In the subpopulation of patients ≥65 years of age, the NIS4^®^ and NIS2+™ mean scores were plotted by category of NAS (0–1, 2–3, 4–5, 6–8) and liver fibrosis stage (F0–F4) (Figure 2). There were significant differences in the NIS4^®^ and NIS2+™ scores between the 4 categories of NAS, highlighting the ability of these tests to stratify patients by the metaboinflammatory component of their liver disease (Figure 2A, B). There were also differences in the NIS4^®^ and NIS2+™ scores across the 4 stages of liver fibrosis, with both tests exhibiting the highest discrimination between stages 1 and 2 (Figure 2C, D).

## DISCUSSION

The current study is the first to show the clinical performance of NIS4^®^, and the improved performance of NIS2+™ compared to NIS4^®^, in a population of older adults (≥65 years of age) with at least 1 metabolic risk factor such as T2D, obesity, dyslipidemia, or hypertension. Based on their AUROC values, both NIS4^®^ and NIS2+™ were able to identify patients with at-risk NASH equally well in patients who were <65 versus those who were ≥65 years. Furthermore, while both NIS4^®^ and NIS2+™ may enable physicians to rule out or rule in at-risk NASH in a well-balanced manner with respect to sensitivity and specificity, NIS2+™ was associated with more robust and stable clinical performance across both age subpopulations. This is important because other blood-based tests for identifying patients with liver fibrosis, such as NFS and FIB-4, have shown differing diagnostic discrimination in older (≥65 y) versus younger (<35 y) adults.^28,36,37^ It has been suggested that for FIB-4, age-dependent cutoffs might be more appropriate.^36,37^ However, age-dependent cutoffs for FIB-4 have not been standardized and were not recommended in the recent AASLD or American Association for Clinical Endocrinology guidelines; therefore, we used the more commonly used FIB-4 cutoffs for this study.^13,38^ Both tests (NIS4^®^ and NIS2+™) had much smaller indeterminate or moderate risk zones relative to other blood tests that were developed for advanced liver fibrosis. The data in this study confirm previously published assay performance data in independent patient cohorts.^28–30^ Of all the blood-based tests that were previously compared, NIS2+™ exhibited the best clinical performance, with the highest AUROC and a more balanced sensitivity and specificity at the low, high, and Youden cutoff points.^31^ The current study extends these observations by showing that NIS2+™ exhibits effective overall performance in a patient population that reflects the Medicare population in the United States. One of the strengths of NIS2+™ is the ability to identify at-risk NASH at a stage where lifestyle modification and/or therapeutic agents can reduce liver inflammation and fibrosis. Noninvasive tests, such as FIB-4 and ELF™, were designed to identify patients with advanced liver fibrosis and a high likelihood of progression to end-stage liver disease. Patients identified using these tests could be treated with drugs designed for reducing advanced liver fibrosis, which is a later disease stage. NIS2+™ test results, on the other hand, may permit physicians to more confidently identify patients earlier in the course of metabolic liver disease and consider prescribing intensive lifestyle or a therapeutic intervention specifically designed to treat the metaboloinflammatory component of NASH in order to reduce progression to end-stage liver disease.

Optimization of the NIS4^®^ technology to produce the NIS2+™ test provided several advantages. As previously published, after optimization of the equation, HbA1c and α2-macroglobulin no longer contributed significantly to the prediction of at-risk NASH; therefore, they were removed from the equation.^31^ Removing HbA1c also avoids the perceived issue that use of antidiabetic medications that reduce HbA1c levels may affect scores and may complicate the interpretation of the test result, especially in patients with uncontrolled HbA1c who require aggressive treatment. The further addition of a sex correction for miR-34a-5p enabled the NIS2+™ results to be independent of sex. Therefore, the NIS2+™ is more balanced than NIS4^®^ with respect to age, sex, and T2D status; however, it retains the strong assay performance for the detection of at-risk NASH. Moreover, the ROC curve (Figure 1) and clinical performance (Table 4) show that NIS2+™ has higher specificity for ruling in at-risk NASH than NIS4^®^; however, it retains high sensitivity for ruling out at-risk NASH. In addition, NIS2+™ appears to better differentiate liver fibrosis stages F2-F4 from stages F0-F1 than NIS4^®^, speaking to the enhanced ability to differentiate patients with at-risk NASH from those with no to mild fibrosis (Figure 2). Current AASLD and American Association for Clinical Endocrinology guidelines support screening of patients with multiple metabolic risk factors and/or T2D for NASH.^13,38^ For the first time, the American Heart Association acknowledged in a published statement that NAFLD is a risk factor for atherosclerotic cardiovascular disease and that CVD is the principal cause of death in patients with NAFLD.^39^ Several recent publications have also been directed toward primary care physicians and endocrinologists in order to increase awareness of the disease as well as provide guidance on how to evaluate and treat patients identified as having NAFLD/NASH.^40–42^ Increased awareness of NAFLD/NASH and adherence to newly published guidelines will likely lead to a reduction in patient progression to cirrhosis, HCC, and liver transplantation. Moreover, the addition of new tests for the identification of patients with at-risk NASH, such as NIS2+™, should facilitate this goal by identifying patients in the primary care or endocrinology setting. By identifying patients earlier in their disease, there may also be a concomitant reduction in health care utilization and costs in a disease that is growing in prevalence yet remains somewhat silent, meaning that it is currently hard to detect until later in a patient’s disease path.

The strengths of this study include the large number of patients who were ≥65 years of age as well as the fact that the study cohort was balanced with respect to the various stages of disease activity and liver fibrosis. In addition, patients were included with or without increased ALT or AST and came from multiple general and specialty practice settings including hepatology, gastroenterology, and internal medicine. These points are especially important because they substantiate the fact that NIS2+™ is able to identify patients with at-risk NASH among individuals with various stages of NAFLD, and could be used as a screening tool in a population of patients with metabolic risk factors.

The limitations to this study include the fact that the RESOLVE-IT diag cohort did not have data for Pro-C3, a biomarker of fibrosis that has been used in other NASH trials, nor did it have sufficient VCTE or magnetic resonance elastography data to compare with the results from the blood-based tests reported here. Because this data set did not have VCTE results for the majority of the participants, and no CAP scores, it was not possible to compare the blood-based tests with combined imaging and blood-based algorithms such as the FAST score.

## CONCLUSIONS

The clinical performance of NIS2+™ was superior to that of NIS4^®^, FIB-4, NFS, ALT, and ELF™ for the identification of at-risk NASH in patients aged ≥65 years. Therefore, these data support the value of this test for the detection of at-risk NASH in older adults.

## AUTHOR CONTRIBUTIONS

Jérémy Magnanensi contributed to the study design, data generation, data analysis, and publication writing. Margery A. Connelly contributed to the study design, data analysis, and publication writing. Arun J. Sanyal, Zouher Majd, Christian Rosenquist, Delphis M. Vera, and James P. Almas contributed to the data analysis and publication writing. All authors reviewed, edited, and approved the final version of the manuscript.

### ACKNOWLEDGMENTS

The authors would like to thank Dr. Bruce Damiano for careful review of the manuscript.

### FUNDING INFORMATION

Arun J. Sanyal received funding from NIH project R01 DK105961.

### CONFLICTS OF INTEREST

Jérémy Magnanensi is employed by GENFIT. Zouher Majd is employed by GENFIT. Christian Rosenquist is employed by GENFIT. Delphis M. Vera is employed by Labcorp. James P. Almas is employed by Labcorp. Margery A. Connelly is employed by Labcorp. Arun J. Sanyal consults and received grants from AstraZeneca, Gilead, Intercept, Merck, Novartis, Salix, and Tobira. He consults and owns stock in GENFIT. He is employed by and owns stock in Sanyal Biotechnology. He consults for Afimmune, Albireo, Chemomab, Conatus, Echosens, Eli Lilly, Exhalenz, Fractyl, Galectin, Hemoshear, Immuron, Janssen, Nimbus, Nitto Denko, Nordic Bioscience, Novo Nordisk, Pfizer, Prosciento, Surrozen, Synlogic, Takeda, Terns, Tobira, Valeant, Zafgen, and Zydus. He received grants from Bristol Myers Squibb, Cumberland, Mallinckrodt, and Shire. He is employed by Virginia Commonwealth University. He owns stock in Akarna, Birdrock, Boehringer-Ingelheim, Bristol Myers Squibb, Durect Inversago, Galmed, Indalo, and Tiziana. He holds intellectual property rights with Elsevier and UpToDate. The remaining authors have nothing to report.

## Supplementary Material

## References

[1] Younossi Z Anstee QM Marietti M Hardy T Henry L Eslam M. Global burden of NAFLD and NASH: trends, predictions, risk factors and prevention. Nat Rev Gastroenterol Hepatol. 2018;15:11–20. 28930295 10.1038/nrgastro.2017.109

[2] Younossi ZM. Non-alcoholic fatty liver disease—a global public health perspective. J Hepatol. 2019;70:531–544. 30414863 10.1016/j.jhep.2018.10.033

[3] Kabarra K Golabi P Younossi ZM. Nonalcoholic steatohepatitis: global impact and clinical consequences. Endocr Connect. 2021;10:R240–7. 34486981 10.1530/EC-21-0048 PMC8558888

[4] Younossi ZM Tampi R Priyadarshini M Nader F Younossi IM Racila A. Burden of illness and economic model for patients with nonalcoholic steatohepatitis in the United States. Hepatology. 2019;69:564–572. 30180285 10.1002/hep.30254

[5] Arshad T Paik JM Biswas R Alqahtani SA Henry L Younossi ZM. Nonalcoholic fatty liver disease prevalence trends among adolescents and young adults in the United States, 2007-2016. Hepatol Commun. 2021;5:1676–1688. 34558817 10.1002/hep4.1760 PMC8485885

[6] Golabi P Paik JM Harring M Younossi E Kabbara K Younossi ZM. Prevalence of high and moderate risk nonalcoholic fatty liver disease among adults in the United States, 1999-2016. Clin Gastroenterol Hepatol. 2021;20. doi: 10.1016/j.cgh.2021.12.015 34929391

[7] Noureddin M Ntanios F Malhotra D Hoover K Emir B McLeod E. Predicting NAFLD prevalence in the United States using National Health and Nutrition Examination Survey 2017-2018 transient elastography data and application of machine learning. Hepatol Commun. 2022;6:1537–1548. 35365931 10.1002/hep4.1935 PMC9234676

[8] Hamid O Eltelbany A Mohammed A Alsabbagh Alchirazi K Trakroo S Asaad I. The epidemiology of non-alcoholic steatohepatitis (NASH) in the United States between 2010-2020: a population-based study. Ann Hepatol. 2022;27:100727. 35700934 10.1016/j.aohep.2022.100727

[9] Sayiner M Arshad T Golabi P Paik J Farhat F Younossi ZM. Extrahepatic manifestations and healthcare expenditures of non-alcoholic fatty liver disease in the Medicare population. Hepatol Int. 2020;14:556–566. 32300995 10.1007/s12072-020-10038-w

[10] Perumpail BJ Khan MA Yoo ER Cholankeril G Kim D Ahmed A. Clinical epidemiology and disease burden of nonalcoholic fatty liver disease. World J Gastroenterol. 2017;23:8263–8276. 29307986 10.3748/wjg.v23.i47.8263 PMC5743497

[11] Estes C Anstee QM Arias-Loste MT Bantel H Bellentani S Caballeria J. Modeling NAFLD disease burden in China, France, Germany, Italy, Japan, Spain, United Kingdom, and United States for the period 2016-2030. J Hepatol. 2018;69:896–904. 29886156 10.1016/j.jhep.2018.05.036

[12] Dulai PS Singh S Patel J Soni M Prokop LJ Younossi Z. Increased risk of mortality by fibrosis stage in nonalcoholic fatty liver disease: Systematic review and meta-analysis. Hepatology. 2017;65:1557–1565. 28130788 10.1002/hep.29085 PMC5397356

[13] Rinella ME Neuschwander-Tetri BA Siddiqui MS Abdelmalek MF Caldwell S Barb D. AASLD practice guidance on the clinical assessment and management of nonalcoholic fatty liver disease. Hepatology. 2023;77. doi:10.1097/HEP.0000000000000323 PMC10735173 36727674

[14] Simon TG Roelstraete B Khalili H Hagström H Ludvigsson JF. Mortality in biopsy-confirmed nonalcoholic fatty liver disease: results from a nationwide cohort. Gut. 2021;70:1375–1382. 33037056 10.1136/gutjnl-2020-322786 PMC8185553

[15] Newsome PN Sasso M Deeks JJ Paredes A Boursier J Chan WK. FibroScan-AST (FAST) score for the non-invasive identification of patients with non-alcoholic steatohepatitis with significant activity and fibrosis: a prospective derivation and global validation study. Lancet Gastroenterol Hepatol. 2020;5:362–373. 32027858 10.1016/S2468-1253(19)30383-8 PMC7066580

[16] Wong RJ Aguilar M Cheung R Perumpail RB Harrison SA Younossi ZM. Nonalcoholic steatohepatitis is the second leading etiology of liver disease among adults awaiting liver transplantation in the United States. Gastroenterology. 2015;148:547–555. 25461851 10.1053/j.gastro.2014.11.039

[17] Younossi ZM. Nonalcoholic fatty liver disease and nonalcoholic steatohepatitis: implications for liver transplantation. Liver Transpl. 2018;24:166–170. 29272073 10.1002/lt.25003

[18] Sayiner M Younossi ZM. Nonalcoholic steatohepatitis is becoming a top indication for liver transplantation worldwide. Liver Transpl. 2019;25:10–11. 30472779 10.1002/lt.25387

[19] Younossi Z Stepanova M Ong JP Jacobson IM Bugianesi E Duseja A. Nonalcoholic steatohepatitis is the fastest growing cause of hepatocellular carcinoma in liver transplant candidates. Clin Gastroenterol Hepatol. 2019;17:748–55. e3. 29908364 10.1016/j.cgh.2018.05.057

[20] Younossi ZM Harring M Younossi Y Ong JP Alqahtani SA Stepanova M. The impact of NASH to liver transplantations with hepatocellular carcinoma in the United States. Clin Gastroenterol Hepatol. 2021;20. doi:10.1016/j.cgh.2021.10.018 34666156

[21] Loomba R Wong R Fraysse J Shreay S Li S Harrison S. Nonalcoholic fatty liver disease progression rates to cirrhosis and progression of cirrhosis to decompensation and mortality: a real world analysis of Medicare data. Aliment Pharmacol Ther. 2020;51:1149–1159. 32372515 10.1111/apt.15679

[22] Sundaram V Jalan R Shah P Singal AK Patel AA Wu T. Acute on chronic liver failure from nonalcoholic fatty liver disease: a growing and aging cohort with rising mortality. Hepatology. 2021;73:1932–1944. 32961608 10.1002/hep.31566

[23] Stepanova M Kabbara K Mohess D Verma M Roche‐Green A AlQahtani S. Nonalcoholic steatohepatitis is the most common indication for liver transplantation among the elderly: data from the United States Scientific Registry of Transplant Recipients. Hepatol Commun. 2022;6:1506–1515. 35224886 10.1002/hep4.1915 PMC9234626

[24] Karim MA Singal AG Kum HC Lee YT Park S Rich NE. Clinical characteristics and outcomes of nonalcoholic fatty liver disease-associated hepatocellular carcinoma in the United States. Clin Gastroenterol Hepatol. 2022;21. doi:10.1016/j.cgh.2022.03.010 PMC9481743 35307595

[25] Hester D Golabi P Paik J Younossi I Mishra A Younossi ZM. Among Medicare Patients With Hepatocellular Carcinoma, Non-alcoholic Fatty Liver Disease is the Most Common Etiology and Cause of Mortality. *J Clin Gastroenterol*. 2020;54:459–467. 30672817 10.1097/MCG.0000000000001172

[26] Younossi ZM Stepanova M Ong J Trimble G AlQahtani S Younossi I. Nonalcoholic steatohepatitis is the most rapidly increasing indication for liver transplantation in the United States. Clin Gastroenterol Hepatol. 2021;19:580–589.e5. 32531342 10.1016/j.cgh.2020.05.064

[27] Gordon S C. Fraysse J Li S Ozbay AB Wong RJ. Disease severity is associated with higher healthcare utilization in nonalcoholic steatohepatitis Medicare patients. Am J Gastroenterol. 2020;115:562–574. 31833859 10.14309/ajg.0000000000000484

[28] Harrison SA Ratziu V Boursier J Francque S Bedossa P Majd Z. A blood-based biomarker panel (NIS4) for non-invasive diagnosis of non-alcoholic steatohepatitis and liver fibrosis: a prospective derivation and global validation study. Lancet Gastroenterol Hepatol. 2020;5:970–985. 32763196 10.1016/S2468-1253(20)30252-1

[29] Sanyal AJ Shankar SS Calle RA Samir AE Sirlin CB Sherlock SP. Non-invasive biomarkers of nonalcoholic steatohepatitis: the FNIH NIMBLE project. Nature Med. 2022;28:430–432. 35145308 10.1038/s41591-021-01652-8 PMC9588405

[30] Sanyal AJ Shankar SS Yates KP Daly E Dehn CA Bolognese JA. Primary results of the NIMBLE stage 1-NASH CRN study of circulating biomarkers for nonalcoholic steatohepatitis and its activity and fibrosis stage. Hepatology. 2021;74:1383A.

[31] Harrison SA Ratziu V Magnanensi J Hajji Y Deledicque S Majd Z. NIS2+™, an optimization of the blood-based biomarker NIS4^®^® technology for the detection of at-risk NASH: a prospective derivation and validation study. J Hepatol. 2023. doi:10.1016/j.jhep.2023.04.031 In press. 37224923

[32] Lichtinghagen R Pietsch D Bantel H Manns MP Brand K Bahr MJ. The Enhanced Liver Fibrosis (ELF) score: normal values, influence factors and proposed cut-off values. J Hepatol. 2013;59:236–342. 23523583 10.1016/j.jhep.2013.03.016

[33] Angulo P Hui JM Marchesini G Bugianesi E George J Farrell GC. The NAFLD fibrosis score: a noninvasive system that identifies liver fibrosis in patients with NAFLD. Hepatology. 2007;45:846–854. 17393509 10.1002/hep.21496

[34] Vallet-Pichard A Mallet V Nalpas B Verkarre V Nalpas A Dhalluin-Venier V. FIB-4: an inexpensive and accurate marker of fibrosis in HCV infection. Comparison with liver biopsy and fibrotest. Hepatology. 2007;46:32–36. 17567829 10.1002/hep.21669

[35] Shah AG Lydecker A Murray K Tetri BN Contos MJ Sanyal AJ. Comparison of noninvasive markers of fibrosis in patients with nonalcoholic fatty liver disease. Clin Gastroenterol Hepatol. 2009;7:1104–1112. 19523535 10.1016/j.cgh.2009.05.033 PMC3079239

[36] McPherson S Hardy T Dufour JF Petta S Romero-Gomez M Allison M. Age as a confounding factor for the accurate non-invasive diagnosis of advanced NAFLD fibrosis. Am J Gastroenterol. 2017;112:740–751. 27725647 10.1038/ajg.2016.453 PMC5418560

[37] Ishiba H Sumida Y Tanaka S Yoneda M Hyogo H Ono M. The novel cutoff points for the FIB4 index categorized by age increase the diagnostic accuracy in NAFLD: a multi-center study. J Gastroenterol. 2018;53:1216–1224. 29744597 10.1007/s00535-018-1474-y

[38] Cusi K Isaacs S Barb D Basu R Caprio S Garvey WT. American Association of Clinical Endocrinology Clinical Practice Guideline for the Diagnosis and Management of Nonalcoholic Fatty Liver Disease in Primary Care and Endocrinology Clinical Settings: Co-Sponsored by the American Association for the Study of Liver Diseases (AASLD). Endocr Pract. 2022;28:528–562. 35569886 10.1016/j.eprac.2022.03.010

[39] Duell PB Welty FK Miller M Chait A Hammond G Ahmad Z. Nonalcoholic fatty liver disease and cardiovascular risk: a scientific statement from the American Heart Association. Arterioscler Thromb Vasc Bio. 2022;42:e168–e185. 35418240 10.1161/ATV.0000000000000153

[40] Wong VWS Zelber‐Sagi S Cusi K Carrieri P Wright E Crespo J. Management of NAFLD in primary care settings. Liver Int. 2022;42:2377–2389. 35986897 10.1111/liv.15404

[41] Cusi K. A diabetologist’s perspective of non-alcoholic steatohepatitis (NASH): knowledge gaps and future directions. Liver Int. 2020;40(suppl 1):82–88. 32077613 10.1111/liv.14350

[42] Cusi K. Time to include nonalcoholic steatohepatitis in the nanagement of patients with type 2 diabetes. Diabetes care. 2020;43:275–279. 31959644 10.2337/dci19-0064

