# Supplementary material for: NIS2+™, an effective blood-based test for the diagnosis of at-risk nonalcoholic steatohepatitis in adults 65 years and older
Source: Hepatol Commun. 2023 Aug 9;7(9):e0223. doi: 10.1097/HC9.0000000000000223 (PMC10412428; doi:10.1097/HC9.0000000000000223)
Supplement: Supplementary file 1 [file hc9-7-e0223-s001.docx]

Supplemental Table 1: Clinical performance of NIS2+^TM^ for the detection of at-risk NASH F≥3 and F4, by age group

|  | **At-risk NASH F≥3 endpoint** | | **At-risk NASH F4 endpoint** | |
| --- | --- | --- | --- | --- |
|  | < 65 years | ≥ 65 years | < 65 years | ≥ 65 years |
| **Prevalence** | 409 (24.9) | 132 (32.2) | 72 (4.4) | 21 (5.1) |
| **AUROC** | 0.78 (0.76, 0.81) | 0.79 (0.75, 0.84) | 0.77 (0.73, 0.81) | 0.70 (0.62, 0.77) |
| **Rule-out (High Risk)** |  |  |  |  |
| Low cutoff | 0.46 | 0.46 | 0.46 | 0.46 |
| Sensitivity | 89.2 (85.7, 92.0) | 91.7 (85.2, 95.6) | 95.8 (87.5, 98.9) | 95.2 (74.1, 99.8) |
| Specificity | 51.5 (48.7, 54.4) | 45.0 (39.0, 51.0) | 43.1 (40.6, 45.6) | 34.7 (30, 39.7) |
| NPV | 93.5 (91.3, 95.2) | 91.9 (85.7, 95.7) | 99.6 (98.6, 99.9) | 99.3 (95.4, 100) |
| **Intermediate zone (Moderate Risk)** | 374 (23) | 101 (25) | 374 (23) | 101 (25) |
| **Rule-in (Low Risk)** |  |  |  |  |
| High cutoff | 0.68 | 0.68 | 0.68 | 0.68 |
| Sensitivity | 69.9 (65.2, 74.3) | 72.7 (64.2, 79.9) | 77.8 (66.2, 86.4) | 76.2 (52.5, 90.9) |
| Specificity | 75.4 (72.9, 77.8) | 72.3 (66.6, 77.4) | 66.1 (63.7, 68.4) | 59.6 (54.6, 64.5) |
| PPV | 48.6 (44.5, 52.7) | 55.5 (47.8, 63.0) | 9.5 (7.3, 12.2) | 9.2 (5.5, 14.8) |

Data are either percentage (95% confidence limits) or n (%) for the Intermediate zone and Prevalence. Abbreviations: AUROC, area under the receiver operating characteristics curve; NASH, non-alcoholic steatohepatitis; NPV, negative predictive value; PPV, positive predictive value.
